# Annotations

**Published:** 1908-10-10

**Authors:** 


### ANNOTATIONS.

### The Endowment of Heroism.

In the preliminary announcement, which was published in the papers last week, of his proposal to establish a "Hero Fund" for this country, Mr. Andrew Carnegie pays a graceful tribute to the medical and nursing professions. "No action," he says, in his letter to the Carnegie Trustees at Dunfermline, "is more heroic than that of doctors and nurses volunteering their services in the case of epidemics. . . . Whenever heroism is displayed by man or woman in saving human life in peaceful pursuits the fund applies." As is already well-known to most of our readers, Mr. Carnegie proposes to devote an endowment yielding an annual income of £12,500 to the purpose of liberating from poverty, or even from pecuniary anxiety, the persons, and the families of persons, by whom deeds of heroism which take the form of endeavours to save human life have been performed. Mr. Carnegie has the satisfaction of knowing that his Majesty the King approves of this munificent project for the endowment of heroism; and under a wise and careful administration the fund should prove to be an encouragement to the performance of heroic and self-sacrificing acts in times of peace. The trustees to whom he commits the management and distribution of the fund have already shown themselves worthy of his confidence, and we feel sure that they will avoid the possible disadvantages which in less capable hands might result from a comprehensive scheme for the pecuniary recognition of heroism. Even those who feel a little doubtful on general principles of the wisdom of establishing such a fund must acknowledge the generosity and broad humanity which have inspired Mr. Carnegie to set apart this large sum of money for the benefit of the unselfish and courageous.

### The Notification of Consumption.

The order of the Local Government Board recently announced by Dr. Newsholme, the lately appointed medical officer of the Board, is a step which, once taken, is not likely to be retracted; and, if it does not deal fully or adequately with the problem concerned, it is yet an instalment of advance along right lines for which we must all be thankful. According to the terms of this order all Poor-law medical officers, whether their patients are under treatment in their homes or in institutions of any kind, are required to notify to the local medical officer of health, should he desire it, all parish patients suffering from tuberculosis; and this he must do within 48 hours of the establishment of the diagnosis. The medical officer of health, it has been pointed out, is not likely to trouble himself to collect this information unless his sanitary authority is able and anxious to support him in the fight against the disease in question; but, wherever he is thus backed up, it is easy to see what a powerful lever the order of the Local Government Board has placed in his hands. Instead of working in the dark against overcrowding, defective sanitation, and the other factors which predispose to tuberculosis in cities, he can direct his energies against the actual foci whence infection is or may be disseminated. A slum tenement becomes a far more menacing danger to the public weal when it harbours tuberculous inmates in its swarming population. The case for the destruction of an unhygienic hovel is comprehensible to the densest of local authorities when a string of notifications can be exhibited from within its walls. More important still, an infected individual can be removed from opportunities of spreading contagion in houses which otherwise are not unsuited to the needs of their inhabitants; and there are other advantages, which medical men can readily picture for themselves, likely to arise from this notification of tuberculosis. Very well, then, says common sense, if this is such a good thing, why not extend the benefits to include the whole community? And medical science can only echo, Why not? We fancy the answer is to be sought in the peculiar British distrust of innovation. The step which Mr. Burns has taken is one which is within his powers as Secretary, and it does not excite the prejudices of anyone who has a vote. It will, therefore, come quietly into operation without much opposition, we may believe, and the gradual demonstration of the utility of the measure may, it is to be hoped, slowly convince the public of the wisdom of its general application. Thus in time the profession may arouse the nation to the serious issues that hang on the war against tuberculosis.

### A Superintendent of Out-patients.

The London Hospital has one of the most completely organised out-patient departments in the world. In that department some 1,300 out-patients are seen and dealt with every day. The work connected with it is enormous, and the authorities have determined to make a wise departure by appointing a special officer, with the title of Superintendent of Out-patients, to take charge. The duties of the superintendent will be to take general charge of the whole department, with authority to report to the secretary any irregularity or unpunctuality in the attendance of those on duty, and anything which he thinks of importance at any time. It will be his duty to check any glaring cases of abuse, secure the proper registration of all patients, and prevent overcrowding. He will also be held responsible for the admission of patients into the wards. In addition to this he will have under him the staff of porters and other employees, and he will be expected to make suggestions with a view to improvement in the general working of the department from time to time. This account of the duties of the new officer points clearly to a determined effort to secure thorough efficiency, and we hope it may lead to the ultimate adoption by the London Hospital of some scheme of filtration on the basis of the Birmingham scheme which is set forth in a later part of this issue.

